# Accumulation of CTCF-binding sites drives expression divergence between tandemly duplicated genes in humans

**DOI:** 10.1186/1471-2164-15-S1-S8

**Published:** 2014-01-24

**Authors:** Ben-Yang Liao, Andrew Ying-Fei Chang

**Affiliations:** Division of Biostatistics & Bioinformatics, Institute of Population Health Sciences, National Health Research Institutes, Zhunan, Miaoli County, 350 Taiwan R.O.C

**Keywords:** epigenetics, DNA methylation, genome architecture, ChIP-seq, RNA-seq

## Abstract

**Background:**

During eukaryotic genome evolution, tandem gene duplication is the most frequent event giving rise to clustered gene families. However, how expression divergence between tandemly duplicated genes has emerged and maintained remain unclear. In particular, it is unknown if epigenetic regulators have been involved in the process.

**Results:**

We demonstrate that CCCTC-binding factor (CTCF), the master epigenetic regulator and the only known insulator protein in humans, has played a predominant role in generating divergence in both expression profiles and expression levels between adjacent paralogs in the human genome. This phenomenon was not observed for non-paralogous adjacent genes. After tandem duplication events, CTCF-binding sites gradually accumulate between paralogs. This trend was more prominent for genes involved in particular functions.

**Conclusions:**

The accumulation of CTCF-binding sites drives expression divergence of tandemly duplicated genes. This process is likely targeted by natural selection. Our study reveals the importance of CTCF to the evolution of animal diversity and complexity.

**Electronic supplementary material:**

The online version of this article (doi:10.1186/1471-2164-15-S1-S8) contains supplementary material, which is available to authorized users.

## Background

Gene duplication is a major driver for the emergence of organismal complexity and evolutionary innovations [1, 2]. Tandem duplication is the most common route to the formation of clustered paralogous genes [3, 4]. A newly duplicated gene must diverge from its progenitor gene in coding sequence or expression, or it will degenerate due to redundancy [1, 5, 6]. To acquire novel transcription patterns, tandemly duplicated genes need to interrupt expression similarity due to shared upstream *cis*-elements upon origin [3, 7] and transcriptional interference contributed by physical proximity [8]. Despite the challenges, functionally important gene clusters consisting of paralogs with distinct expression patterns are found in a wide range of species, including human [9, 10]. Therefore, it is important to understand the origin and maintenance of expression divergence between tandem paralogs.

CCCTC-binding factor (CTCF), the only known human insulator protein, plays a master role in determining the transcriptional landscape of genomes. When bound to insulator sequences (CTCF-binding sites), CTCF can prevent repressive heterochromatin from spreading into neighboring regions [11, 12]. In addition, CTCF interferes with enhancer-promoter communication [13] and guides long-range chromatin interactions [14]. Because changes in epigenetic marks play roles in regulatory divergence of duplicated genes [15–17], we hypothesize that CTCF also plays a role. Using human RNA-seq and ChIP-seq data (see Methods), we examined how CTCF drives regulatory divergence of duplicated genes, especially tandemly arrayed paralogs.

## Results and discussion

In the genome, adjacent genes are coexpressed due to a common origin [18], cofunctionality [19], or deleterious transcriptional interference [8]. Using RNA-seq data from six human adult tissues [20], we measured gene expression dissimilarity between adjacent genes in terms of expression profile or expression level using *ExpD*_1-r_ or *ExpD*_Euc_ (see Methods), respectively. *ExpD*_1-r_ focused on changes in the shape across the tissue dimension, while *ExpD*_Euc_ focused on summed changes in abundance. We studied divergences in both profiles and abundances because previous studies have shown that the observations from the two aspects did not necessarily produce consistent results [21], possibly due to different underlying mechanisms controlling the properties [22].

Mammalian genes close to each other have similar expression profiles [8, 19]. CTCF-binding sites can prevent undesirable crosstalk between active and inactive genomic regions [23]. DNA methylation upstream of a gene can inhibit its transcriptional initiation [24]. To determine if the effect of intergenic distance, the number of CTCF-binding sites, and DNA methylation is related to shared evolutionary origin, we measured *ExpD*_1-r_ and *ExpD*_Euc_ in non-paralogous adjacent genes. Non-paralogous gene pairs with a longer intergenic distance (*d*, calculated as the distance in nucleotides between the transcriptional start sites, TSS), more overlapping CTCF-binding sites (*#CTCF*, see Methods), or a larger difference in upstream DNA methylation (Δ*CpG*_O/E_, see Methods) had greater *ExpD*_1-r_ or *ExpD*_Euc_ (see ρ in Table 1). Although *d*, *#CTCF*, and Δ*CpG*_O/E_ were interrelated (*d* vs. *#CTCF*: ρ=0.521, *P*<10^-300^; *#CTCF* vs. Δ*CpG*_O/E_: ρ=0.075, *P*<10^-40^; Δ*CpG*_O/E_ vs. *d*: ρ=0.166, *P*<10^-192^), partial correlation analyses (see Methods) suggested that the effects of *d*, *#CTCF*, and Δ*CpG*_O/E_ on generating expression dissimilarity between adjacent genes was independent. *d* had the largest direct influence on *ExpD*_1-r,_ and Δ*CpG*_O/E_ had the largest direct influence on *ExpD*_Euc_. *#CTCF* had a weak but significant effect on *ExpD*_1-r_ and an intermediate effect on *ExpD*_Euc_ (see ρ_p_ in Table 1). Although CTCF binding can vary among cell types [25], when we define *#CTCF* using joint CTCF ChIP-seq peaks (see Methods) instead of overlapping CTCF peaks, the results did not change (Table S1 in Additional file 1).

**Table 1:** Rank correlations (ρ) and partial rank correlations (ρ_p_) of the genomic properties with expression dissimilarities (measured by *ExpD*_1-__r_ or *ExpD*_Euc_) of the non-paralogous adjacent genes

Genomic properties ^a^	***ExpD***_1-r_		***ExpD***_Euc_	
	**ρ (** ***P*** **-value)** ^**b**^	**ρ**_**p**_ **(** ***P*** **-value)** ^**b,c**^	**ρ (** ***P*** **-value)** ^**b**^	**ρ**_**p**_ **(** ***P*** **-value)** ^**b,c**^
*d*	0.180 (<10^-217^)	0.130 (<10^-113^)	0.112 (<10^-83^)	0.040 (<10^-11^)
*#CTCF*	0.120 (<10^-98^)	0.032 (<10^-7^)	0.104 (<10^-72^)	0.058 (<10^-22^)
Δ*CpG*_O/E_	0.077 (<10^-39^)	0.049 (<10^-16^)	0.196 (<10^-258^)	0.182 (<10^-227^)

^a^ "*d*", intergenic distance; "*#CTCF*", number of overlapping CTCF-binding sites; "*ΔCpG*_O/E_", difference in upstream DNA methylation.

^b^ *P* values show the probabilities of the observations under the hypothesis of no correlation.

^c^ Spearman's partial correlation coefficient ρ_p_ is computed by controlling for the other two genomic properties listed in ^a^

Mechanisms generating coding sequence divergence, such as change in protein structure or splicing, between paralogous genes have been intensively investigated [2, 26–28]. By contrast, mechanisms generating expression divergence have garnered less attention. Although *#CTCF* was not the strongest determinant of expression divergence in non-paralogous adjacent gene pairs (Table 1), for adjacent paralogs, *#CTCF* had the greatest direct influence on *ExpD*_1-r_ and *ExpD*_Euc_, followed by Δ*CpG*_O/E_ (see ρ_p_ in Table 2). Repeating analysis based on an independently generated and unpublished RNA-seq dataset including 16 human tissues (Illumina BodyMap 2.0 project, see Methods) produced a result consistent with Table 1 and 2 (Table S2 in Additional file 1), suggesting the robustness of the pattern found.

**Table 2:** Rank correlations (ρ) and partial rank correlations (ρ_p_) of the examined genomic properties with expression dissimilarities (measured by *ExpD*_1-__r_ or *ExpD*_Euc_) of the paralogous adjacent genes

Genomic properties ^a^	***ExpD***_1-r_		***ExpD***_Euc_	
	**ρ (** ***P*** **-value)** ^**b**^	**ρ**_**p**_ **(** ***P*** **-value)** ^**b,c**^	**ρ (** ***P*** **-value)** ^**b**^	**ρ**_**p**_ **(** ***P*** **-value)** ^**b,c**^
*d*	0.039 (0.164)	-0.011 (0.707)	0.100 (<10^-3^)	0.021 (0.461)
*#CTCF*	0.111 (<10^-4^)	0.103 (<10^-3^)	0.187 (<10^-10^)	0.159 (<10^-7^)
Δ*CpG*_O/E_	0.069 (0.015)	0.054 (0.023)	0.102 (<10^-3^)	0.094 (<10^-3^)

^a^ "*d*", intergenic distance; "*#CTCF*", number of overlapping CTCF-binding sites; "*ΔCpG*_O/E_", difference in upstream DNA methylation.

^b^ *P* values show the probabilities of the observations under the hypothesis of no correlation.

^c^ Spearman's partial correlation coefficient ρ_p_ is computed by controlling for the other two genomic properties listed in ^a^

We classified adjacent paralogs into three groups according the orientations: head-to-head, head-to-tail and tail-to-tail [29]. Although reduced sample sizes resulted in reduced statistical significance, the pattern of the greatest impact *#CTCF* on expression divergences held regardless of the orientation of paralogs (Table 3). Using microarray expression data, a previous study found that intergenic distance was related to expression divergence for tandemly arrayed paralogs [30]. However, microarray data is known to have cross-hybridization related biases [17], and we found no direct association between intergenic distance and expression divergence for tandemly arrayed paralogs after controlling for *#CTCF* and Δ*CpG*_O/E_ (see ρ_p_ of *d* vs. *ExpD*_1-r_ or *ExpD*_Euc_, Table 2). Taken together, these results suggest that CTCF-binding sites play a very significant, if not primary, role in driving expression divergence of tandemly duplicated genes.

**Table 3:** Rank correlations (ρ) and partial rank correlations (ρ_p_) of the examined genomic properties with expression dissimilarities (measured by *ExpD*_1-__r_ or *ExpD*_Euc_) of the adjacent paralogous genes of different orientations.

Genomic properties ^a^	***ExpD***_1-r_		***ExpD***_Euc_	
	**ρ (** ***P*** **-value)** ^**b**^	**ρ**_**p**_ **(** ***P*** **-value)** ^**b,c**^	**ρ (** ***P*** **-value)** ^**b**^	**ρ**_**p**_ **(** ***P*** **-value)** ^**b,c**^
**Head-to-Head** ^d^				
*d*	0.165 (0.072)	0.106 (0.250)	0.150 (0.109)	0.036 (0.700)
*#CTCF*	0.213 (0.020)	0.173 (0.058)	0.352 (<10^-4^)	0.327 (<10^-3^)
Δ*CpG*_O/E_	-0.024 (0.794)	0.001 (0.991)	-0.071 (0.443)	-0.054 (0.561)
**Head-to-Tail** ^d^				
*d*	0.005 (0.870)	-0.050 (0.110)	0.089 (<10^-2^)	0.016 (0.607)
*#CTCF*	0.100 (<10^-2^)	0.110 (<10^-3^)	0.151 (<10^-5^)	0.127 (<10^-4^)
Δ*CpG*_O/E_	0.083 (<10^-2^)	0.085 (<10^-2^)	0.109 (<10^-3^)	0.104 (<10^-3^)
**Tail-to-Tail** ^d^				
*d*	0.139 (0.110)	0.105 (0.230)	0.145 (0.096)	0.046 (0.602)
*#CTCF*	0.113 (0.195)	0.061 (0.482)	0.271 (<10^-2^)	0.202 (0.019)
Δ*CpG*_O/E_	0.050 (0.569)	0.018 (0.0835)	0.212 (0.014)	0.153 (0.078)

^a^ "*d*", intergenic distance; "*#CTCF*", number of overlapping CTCF-binding sites; "*ΔCpG*_O/E_", difference in upstream DNA methylation.

^b^ *P* values show the probabilities of the observations under the hypothesis of no correlation.

^c^ Spearman's partial correlation coefficient ρ_p_ is computed by controlling for the other two genomic properties listed in ^a^

^d^ There were 120, 1003 and 133 head-to-head, head-to-tail and tail-to-tail adjacent paralogs, respectively.

There are two hypotheses to explain the influence of CTCF-binding sites in driving expression divergence of tandem paralogs. First, tandem paralogs that arose in genomic regions with high densities of CTCF-binding sites nearby are more likely to be preserved due to immediate independence of gene regulation. Second, CTCF-binding sites accumulated over time to enhance independent gene regulation of tandem paralogs, especially those have been functionally diverged. If the first hypothesis is correct, there would be no correlation between the divergence time of paralogs (measured by *d*_S_ or *T*_phy_, see Methods) and *#CTCF*. However, we observed a positive correlation between *d*_S_ or *T*_phy_ and *#CTCF* (Table 4), suggesting that the second hypothesis is correct. *#CTCF* had stronger rank correlation (ρ), which is positive, to *d*_S_ (or *T*_phy_) than *d* and Δ*CpG*_O/E_ (Table 4). Partial correlation analyses further suggested that the increase of *#CTCF* with *d*_S_ (or *T*_phy_) is not caused by the change in *d* or Δ*CpG*_O/E_ or their combined effect over time (see ρ_p_ in Table 4). By contrast, the insignificant partial correlation of *d* with respect to *d*_S_ (or *T*_phy_) indicated that the increase *d* over time can be explained by the increase in the number of CTCF-binding sites and the associated changes in DNA methylation.

**Table 4:** Rank correlations (ρ) and partial rank correlations (ρ_p_) of the examined genomic properties with the divergence time of the paralogous adjacent genes.

Genomic properties ^a^	***d***_S_		***T***_phy_	
	**ρ (** ***P*** **-value)** ^**b**^	**ρ**_**p**_ **(** ***P*** **-value)** ^**b,c**^	**ρ (** ***P*** **-value)** ^**b**^	**ρ**_**p**_ **(** ***P*** **-value)** ^**b,c**^
**All adjacent paralogs**				
*d*	0.138 (<10^-5^)	0.057 (0.056)	0.141 (<10^-6^)	0.053 (0.060)
*#CTCF*	0.225 (<10^-13^)	0.189 (<10^-9^)	0.219 (<10^-14^)	0.178 (<10^-9^)
Δ*CpG*_O/E_	0.097 (<10^-2^)	0.089 (<10^-2^)	0.084 (<10^-2^)	0.073 (<10^-2^)
**Adjacent paralogs associated with GO terms in which high** ***#CTCF/d*** **genes were specifically enriched**				
*d*	0.086 (0.152)	-0.070 (0.245)	0.108 (0.072)	-0.038 (0.526)
*#CTCF*	0.292 (<10^-6^)	0.276 (<10^-5^)	0.273 (<10^-5^)	0.243 (<10^-4^)
Δ*CpG*_O/E_	0.122 (0.043)	0.079 (0.186)	0.132 (0.027)	0.092 (0.125)

^a^ "*d*", intergenic distance; "*#CTCF*", number of overlapping CTCF-binding sites; "*ΔCpG*_O/E_", difference in upstream DNA methylation.

^b^ *P* values show the probabilities of the observations under the hypothesis of no correlation.

^c^ Spearman's partial correlation coefficient ρ_p_ is computed by controlling for the other two genomic properties listed in ^a^

To determine whether tandem paralogs with specific functions tend to have a greater number of intervening CTCF-binding sites, we performed enrichment analyses in Gene Ontology (GO) terms. To eliminate the potential effect of duplicability in GO analysis [4], paralogous and non-paralogous gene pairs were analyzed separately. To control for the potential effect of gene density [31], *#CTCF/d* was used instead of *#CTCF* (although using *#CTCF* produced a consistent result, which is not shown). Gene pairs in the top quartile of CTCF-binding site density (*#CTCF/d*) were compared against the bottom three quartiles. Enriched/depleted GO terms for the paralog group were substantially different from those in the non-paralog group (Table 5). Only two GO terms (GO:0010033, response to organic substance; GO:0031012, extracellular matrix) had the same enrichment status ("enriched") in both groups. Tandemly duplicated genes with a higher density of intervening CTCF-binding sites tended to specifically encode proteins involved in gene expression (GO:0010467, GO:0008134), metabolic processes (GO:0019222, GO:0006139, GO:0050790), or cellular processes (GO:0050794, GO:0051128, GO:0044249) through DNA binding (GO:0003677), SMAD binding (GO:0046332), growth factor binding (GO:0019955), or kinase interaction (GO:0019210, GO:0019887) in receptor complexes (GO:0043235) or intracellular regions (GO:0031012) (Table 5). This result implied that the densities of CTCF binding sites between tandem paralogs were not contributed by the genomic background.

**Table 5:** Enriched/depleted GO terms at level 4 for genes with high *#CTCF/d* flanking regions

Functional categories	Duplicate with 25% top ***#CTCF/d*** vs. rest of duplicates	Non-duplicates with 25% top ***#CTCF/d*** vs. rest of non-duplicates
	**GO terms** ^**a**^ **(↑ or ↓** ^**b**^ **,** ***P*** **-value** ^**c**^ **)**	**GO terms (↑ or ↓** ^**b**^ **,** ***P*** **-value** ^**c**^ **)**
**Molecular functions**		
	**DNA binding** (GO:0003677) (↑, *P*<0.05)	transferase activity, transferring phosphorus-containing groups (GO:0016772) (↑, *P*<10^-7^)
	**transcription factor binding** (GO:0008134) (↑, *P*<10^-2^)	hydrolase activity, acting on acid anhydrides (GO:0016817) (↑, *P*<10^-4^)
	**SMAD binding** (GO:0046332) (↑, *P*<0.05)	protein domain specific binding (GO:0019904) (↑, *P*<10^-2^)
	**growth factor binding** (GO:0019838) (↑, *P*<0.05)	substrate-specific transmembrane transporter activity (GO:0022891) (↑, *P*<10^-4^)
	**kinase inhibitor activity** (GO:0019210) (↑, *P*<0.05)	cytoskeletal protein binding (GO:0008092) (↑, *P*<10^-7^)
	**peptidase inhibitor activity** (GO:0030414) (↑, *P*<0.05)	phospholipid binding (GO:0005543) (↑, *P*<10^-2^)
	**protein kinase regulator activity** (GO:0019887) (↑, *P*<0.05)	identical protein binding (GO:0042802) (↑, *P*<10^-8^)
	**transferase activity, transferring acyl groups** (GO:0016746) (↑, *P*<0.05)	receptor binding (GO:0005102) (↑, *P*<10^-9^)
	transferase activity, transferring glycosyl groups (GO:0016757) (↓, *P*<0.05)	heat shock protein binding (GO:0031072) (↑, *P*<0.05)
	monooxygenase activity (GO:0004497) (↓, *P*<0.05)	oxidoreductase activity, acting on peroxide as acceptor (GO:0016684) (↑, *P*<10^-2^)
**Cellular components**		
	extracellular matrix (GO:0031012) (↑, *P*<10^-6^)	extracellular matrix (GO:0031012) (↑, *P*<10^-3^)
	**intracellular** (GO:0031012) (↑, *P*<0.05)	extracelluar space (GO:0005615) (↑, *P*<10^-8^)
	**receptor complex** (GO:0043235) (↑, *P*<0.05)	endomembrane system (GO:0012505) (↑, *P*<10^-3^)
		membrane-bounded vesicle (GO:0031988) (↑, *P*<0.05)
		cell surface (GO:0009986) (↑, *P*<10^-4^)
		cell projection (GO:0042995) (↑, *P*<0.05)
		midbody (GO:0030496) (↑, *P*<0.05)
**Biological processes**		
	response to organic substance (GO:0010033) (↑, *P*<0.05)	response to organic substance (GO:0010033) (↑, *P*<10^-5^)
	**regulation of metabolic process** (GO:0019222) (↑, *P*<10^-3^)	lipid metabolic process (GO:0006629) (↑, *P*<10^-6^)
	**regulation of catalytic activity** (GO:0050790) (↑, *P*<10^-2^)	defense response (GO:0006952) (↑, *P*<10^-3^)
	**regulation of cellular process** (GO:0050794) (↑, *P*<10^-3^)	response to other organism (GO:0051707) (↑, *P*<0.05)
	**regulation of cellular component organization** (GO:0051128) (↑, *P*<0.05)	carbohydrate metabolic process (GO:0005975) (↑, *P*<0.05)
	**nucleobase, nucleoside, nucleotide and nucleic acid metabolic process** (GO:0006139) (↑, *P*<10^-2^)	response to drug (GO:0042493) (↑, *P*<10^-3^)
	**macromolecule biosynthetic process** (GO:0009059) (↑, *P*<10^-3^)	cell-cell signaling (GO:0007267) (↑, *P*<10^-2^)
	**gene expression** (GO:0010467) (↑, *P*<10^-2^)	cytoskeleton organization (GO:0007010) (↑, *P*<10^-3^)
	**cellular biosynthetic process** (GO:0044249) (↑, *P*<10^-4^)	regulation of developmental process (GO:0050793) (↑, *P*<10^-4^)
	**programmed cell death** (GO:0012501) (↑, *P*<10^-2^)	regulation of immune system process (GO:0002682) (↑, *P*<10^-4^)

^a^ Specifically enriched GO terms are highlighted in bold fonts

^b^ The symbol ↑or ↓ represents the enrichment status of "enriched" or "depleted" of the GO term, respectively.

^c^ *P* values were Bonferroni-corrected for multiple tests

Genomic regions of high densities of CTCF binding sites can emerge through stochastic evolutionary processes. To examine if high CTCF-binding site density between tandem duplicates is the outcome of gradual CTCF-binding site accumulation by natural selection, we focused on the subset of 278 paralogous pairs in which at least one paralog had one of the abovementioned enriched GO categories. In this subset of tandem paralogs, ρ_p_ of *#CTCF* vs. *d*_S_ (or *T*_phy_) after controlling for *d* and Δ*CpG*_O/E_ was stronger (*#CTCF* vs. *d*_S_: ρ_p_=0.276, *P*<10^-5^; *#CTCF* vs. *T*_phy_: ρ_p_=0.243, *P*<10^-4^) than that observed in the full set of adjacent paralogs (*#CTCF* vs. *d*_S_: ρ_p_=0.189, *P*<10^-9^; *#CTCF* vs. *T*_phy_: ρ_p_=0.178, *P*<10^-9^) (Table 4). When *#CTCF* was defined by joint CTCF ChIP-seq peaks rather than overlapping CTCF peaks, the results were similar (Table S3 in Additional file 1). Therefore, the trend to accumulate CTCF-binding sites following a tandem duplication event resulted in more CTCF-binding sites (both absolute number and density) between paralogs, especially for those with enriched GO-categories shown in Table 5.

## Conclusions

Combining human genomic and transcriptomic data, this study demonstrates that CTCF and its binding sites play a major role driving the expression evolution of tandemly duplicated genes. Following tandem duplication events, CTCF-binding sites gradually accumulate between the paralogs to increase their divergences in expression profile and their divergences in expression level. The role of CTCF-binding sites is not limited to the insulation of DNA methylation domains, because the effects of *#CTCF* on *ExpD*_1-r_ (or *ExpD*_Euc_) were still significant even after controlling for Δ*CpG*_O/E_ (Table 2). Thus, CTCF, a conserved regulatory protein [32], affects the expression evolution of adjacent genes in genomes from flies [33] to humans and is important for the evolution of organismal complexity in animals.

## Methods

Annotation of the human genome (Ensembl v72), including gene coordinates, TSS, and paralog divergence, was retrieved through BioMart (http://www.biomart.org/) [34]. Orientation for each paralogous pair (head-to-head, head-to-tail, or tail-to-tail) was determined using strand information. For each paralogous pair, the rate of synonymous changes (*d*_S_) was calculated using PAML [35], and the phylogenetic age (*T*_phy_) was assigned according to Table S4 in Additional file 1 based on Ensembl's annotation of the most recent common ancestor [36]. A smaller *d*_S_ or *T*_phy_ indicated a more recent divergence time. The RNA-seq-based gene expression signals in the human brain, cerebellum, heart, kidney, liver and testis (GSE30352) [20] were obtained from NCBI Gene Expression Omnibus (GEO) (http://www.ncbi.nlm.nih.gov/geo/). The raw reads of RNA-seq data in 16 human tissues (adipose, adrenal, brain, breast, colon, heart, kidney, liver, lung, lymph node, ovary, prostate, skeletal muscle, testes, thyroid, white blood cells) by Illumina BodyMap 2.0 project were downloaded from GSE30611 of GEO and were processed following our previous studies [17, 37] to obtain expression signals. Upstream DNA methylation for a gene was calculated based on the 500 nucleotides upstream of the TSS by CpG_O/E_=*P*_CpG_ /(*P*_C_ × *P*_G_), where *P*_CpG_, *P*_C_, and *P*_G_ are the frequencies of CpG dinucleotides, C nucleotides, and G nucleotides, respectively [17, 38]. The difference in upstream DNA methylation, Δ*CpG*_O/E_, was calculated by the absolute value of the difference in CpG_O/E_ between the two adjacent genes compared. In total, we obtained 30,164 non-paralogous and 1,256 paralogous gene pairs based on 32,164 human genes with detectable expression and estimable CpG_O/E_. *ExpD*_1-r_, which represents the dissimilarity in expression profile, was calculated by 1-Pearson's correlation coefficient of the expression signals of the six tissues. *ExpD*_Euc_, which represents the summed difference in expression levels, was calculated by the Euclidean distance of the expression signals of the six tissues. CTCF-binding sites identified by ChIP-seq experiments [25] in 13 non-cancerous human cells (Table S5 in Additional file 1) were obtained from broadPeak data deposited as GSE30263 at NCBI GEO. We generated two sets of CTCF-binding sites. The number of overlapping CTCF-binding sites, which are CTCF-binding regions present in all examined cell types (Table S5 in Additional file 1), was determined using BEDTools [39]. The number of joint CTCF-binding sites, the union of CTCF-binding sites from all cell types, was determined using a custom Perl script. Enrichment analyses on GO terms were performed using FatiGO [40]. Partial correlation analyses were performed using modules of the "ppcor" package (v.1.0) [41] for R (http://www.r-project.org/).

## Electronic supplementary material

Additional file 1: **Supplementary tables S1-S5**. (DOCX 29 KB)

## References

[1] Ohno S (1970). Evolution by gene duplication.

[2] Zhang J (2003). Evolution by gene duplication: an update. Trends Ecol Evol.

[3] Fan C, Chen Y, Long M (2008). Recurrent tandem gene duplication gave rise to functionally divergent genes in Drosophila. Mol Biol Evol.

[4] Shoja V, Zhang L (2006). A roadmap of tandemly arrayed genes in the genomes of human, mouse, and rat. Mol Biol Evol.

[5] Lynch M, Conery JS (2000). The evolutionary fate and consequences of duplicate genes. Science.

[6] Qian W, Liao B-Y, Chang AY, Zhang J (2010). Maintenance of duplicate genes and their functional redundancy by reduced expression. Trends Genet.

[7] Li W-H, Yang J, Gu X (2005). Expression divergence between duplicate genes. Trends Genet.

[8] Liao B-Y, Zhang J (2008). Coexpression of linked genes in Mammalian genomes is generally disadvantageous. Mol Biol Evol.

[9] Li SS, O'Brien DA, Hou EW, Versola J, Rockett DL, Eddy EM (1989). Differential activity and synthesis of lactate dehydrogenase isozymes A (muscle), B (heart), and C (testis) in mouse spermatogenic cells. Biology of reproduction.

[10] Carroll SB (1995). Homeotic genes and the evolution of arthropods and chordates. Nature.

[11] Cuddapah S, Jothi R, Schones DE, Roh TY, Cui K, Zhao K (2009). Global analysis of the insulator binding protein CTCF in chromatin barrier regions reveals demarcation of active and repressive domains. Genome Res.

[12] Valenzuela L, Kamakaka RT (2006). Chromatin insulators. Annu Rev Genet.

[13] Bell AC, West AG, Felsenfeld G (1999). The protein CTCF is required for the enhancer blocking activity of vertebrate insulators. Cell.

[14] Handoko L, Xu H, Li G, Ngan CY, Chew E, Schnapp M, Lee CW, Ye C, Ping JL, Mulawadi F (2011). CTCF-mediated functional chromatin interactome in pluripotent cells. Nat Genet.

[15] Zou Y, Su Z, Huang W, Gu X (2012). Histone modification pattern evolution after yeast gene duplication. BMC Evol Biol.

[16] Berke L, Sanchez-Perez GF, Snel B (2012). Contribution of the epigenetic mark H3K27me3 to functional divergence after whole genome duplication in Arabidopsis. Genome Biol.

[17] Chang AY, Liao B-Y (2012). DNA methylation rebalances gene dosage after Mammalian gene duplications. Mol Biol Evol.

[18] Lercher MJ, Blumenthal T, Hurst LD (2003). Coexpression of neighboring genes in Caenorhabditis elegans is mostly due to operons and duplicate genes. Genome Res.

[19] Hurst LD, Pal C, Lercher MJ (2004). The evolutionary dynamics of eukaryotic gene order. Nat Rev Genet.

[20] Brawand D, Soumillon M, Necsulea A, Julien P, Csardi G, Harrigan P, Weier M, Liechti A, Aximu-Petri A, Kircher M (2011). The evolution of gene expression levels in mammalian organs. Nature.

[21] Glazko G, Mushegian A (2010). Measuring gene expression divergence: the distance to keep. Biology direct.

[22] Bernstein BE, Birney E, Dunham I, Green ED, Gunter C, Snyder M (2012). An integrated encyclopedia of DNA elements in the human genome. Nature.

[23] Herold M, Bartkuhn M, Renkawitz R (2012). CTCF: insights into insulator function during development. Development.

[24] Jones PA, Takai D (2001). The role of DNA methylation in mammalian epigenetics. Science.

[25] Wang H, Maurano MT, Qu H, Varley KE, Gertz J, Pauli F, Lee K, Canfield T, Weaver M, Sandstrom R (2012). Widespread plasticity in CTCF occupancy linked to DNA methylation. Genome Res.

[26] Hughes AL (1999). Adaptive evolution of genes and genomes.

[27] Su Z, Wang J, Yu J, Huang X, Gu X (2006). Evolution of alternative splicing after gene duplication. Genome Res.

[28] Yandell M, Mungall CJ, Smith C, Prochnik S, Kaminker J, Hartzell G, Lewis S, Rubin GM (2006). Large-scale trends in the evolution of gene structures within 11 animal genomes. PLoS Comput Biol.

[29] Woo YH, Li W-H (2011). Gene clustering pattern, promoter architecture, and gene expression stability in eukaryotic genomes. Proc Natl Acad Sci USA.

[30] Shoja V, Murali TM, Zhang L (2007). Expression divergence of tandemly arrayed genes in human and mouse. Comparative and functional genomics.

[31] Caron H, van Schaik B, van der Mee M, Baas F, Riggins G, van Sluis P, Hermus MC, van Asperen R, Boon K, Voute PA (2001). The human transcriptome map: clustering of highly expressed genes in chromosomal domains. Science.

[32] Moon H, Filippova G, Loukinov D, Pugacheva E, Chen Q, Smith ST, Munhall A, Grewe B, Bartkuhn M, Arnold R (2005). CTCF is conserved from Drosophila to humans and confers enhancer blocking of the Fab-8 insulator. Embo Rep.

[33] Ni X, Zhang YE, Negre N, Chen S, Long M, White KP (2012). Adaptive evolution and the birth of CTCF binding sites in the Drosophila genome. PLoS Biol.

[34] Durinck S, Moreau Y, Kasprzyk A, Davis S, De Moor B, Brazma A, Huber W (2005). BioMart and Bioconductor: a powerful link between biological databases and microarray data analysis. Bioinformatics.

[35] Yang Z (2007). PAML 4: phylogenetic analysis by maximum likelihood. Mol Biol Evol.

[36] Vilella AJ, Severin J, Ureta-Vidal A, Heng L, Durbin R, Birney E (2009). EnsemblCompara GeneTrees: Complete, duplication-aware phylogenetic trees in vertebrates. Genome Res.

[37] Chang TY, Liao BY (2013). Flagellated algae protein evolution suggests the prevalence of lineage-specific rules governing evolutionary rates of eukaryotic proteins. Genome Biol Evol.

[38] Matsuo K, Clay O, Takahashi T, Silke J, Schaffner W (1993). Evidence for erosion of mouse CpG islands during mammalian evolution. Somatic cell and molecular genetics.

[39] Quinlan AR, Hall IM (2010). BEDTools: a flexible suite of utilities for comparing genomic features. Bioinformatics.

[40] Al-Shahrour F, Diaz-Uriarte R, Dopazo J (2004). FatiGO: a web tool for finding significant associations of Gene Ontology terms with groups of genes. Bioinformatics.

[41] Kim SH, Yi SV (2007). Understanding relationship between sequence and functional evolution in yeast proteins. Genetica.

