# Supplementary material for: Accumulation of CTCF-binding sites drives expression divergence between tandemly duplicated genes in humans
Source: BMC Genomics. 2014 Jan 24;15(Suppl 1):S8. doi: 10.1186/1471-2164-15-S1-S8 (PMC4046690; doi:10.1186/1471-2164-15-S1-S8)
Supplement: Supplementary file 1 — Additional file 1: Supplementary tables S1-S5. (DOCX 29 KB) [file 12864_2014_5681_MOESM1_ESM.docx]

**Additional file 1**

**Supplementary Tables and Captions**

**Table S1.** Rank correlations (ρ) and partial rank correlations (ρ_p_) of the examined gene properties with expression dissimilarities of the non-paralogous adjacent genes. *#CTCF* was redefined by joint ChIP-seq peaks.

**Table S2.** Rank correlations (ρ) and partial rank correlations (ρ_p_) of the examined genomic properties with expression dissimilarities of the adjacent genes (*ExpD*_1-r_), which were calculated based on unpublished 16 human tissues of Illumina BodyMap 2.0 project (GSE30611).

**Table S3.** Rank correlations (ρ) and partial rank correlations (ρ_p_) of the examined genomic properties with the divergence time of the adjacent paralogous genes. *#CTCF* was redefined by joint ChIP-seq peaks.

**Table S4.** Assignment of *T*_phy_

**Table S5.** The list of non-cancerous cells from which CTCF-binding sites of the human genome identified by ChIP-seq

**Table S1.** Rank correlations (ρ) and partial rank correlations (ρ_p_) of the examined gene properties with expression dissimilarities (measured by *ExpD*_1-r_ or *ExpD*_Euc_) of the non-paralogous adjacent genes. *#CTCF* was redefined by joint ChIP-seq peaks.

| Genomic properties ^a^ | *ExpD*_1-r_ | | |  | *ExpD*_Euc_ | | |
| --- | --- | --- | --- | --- | --- | --- | --- |
|  | ρ (*P*-value) ^b^ |  | ρ_p_ (*P*-value) ^b,c^ |  | ρ (*P*-value) ^b^ |  | ρ_p_ (*P*-value) ^b,c^ |
| *d* | 0.180 (<10^-217^) |  | 0.075 (<10^-38^) |  | 0.112 (<10^-83^) |  | -0.046 (<10^-15^) |
| *#CTCF* | 0.162 (<10^-177^) |  | 0.021 (<10^-3^) |  | 0.149 (<10^-149^) |  | 0.107 (<10^-77^) |
| ∆*CpG*_O/E_ | 0.077 (<10^-39^) |  | 0.048 (<10^-16^) |  | 0.196 (<10^-258^) |  | 0.183 (<10^-229^) |

^a^ “*d*”, intergenic distance; “*#CTCF*”, number of joint CTCF-binding sites; “*∆CpG*_O/E_”, difference in upstream DNA methylation.

^b^ *P* values show the probabilities of the observations under the hypothesis of no correlation.

^c^ Spearman’s partial correlation coefficient ρ_p_ is computed by controlling for the other two genomic properties listed in ^a^

**Table S2.** Rank correlations (ρ) and partial rank correlations (ρ_p_) of the examined genomic properties with expression dissimilarities of the adjacent genes (*ExpD*_1-r_), which were calculated based on unpublished 16 human tissues of Illumina BodyMap 2.0 project (GSE30611).

| Genomic properties ^a^ | *ExpD*_1-r_ of non-paralogs | | |  | *ExpD*_1-r_ of paralogs | | |
| --- | --- | --- | --- | --- | --- | --- | --- |
|  | ρ (*P*-value) ^b^ |  | ρ_p_ (*P*-value) ^b,c^ |  | ρ (*P*-value) ^b^ |  | ρ_p_ (*P*-value) ^b,c^ |
| *d* | 0.145 (<10^-182^) |  | 0.093 (<10^-75^) |  | 0.083 (<10^-2^) |  | -0.076 (<10^-2^) |
| *#CTCF* | 0.102 (<10^-90^) |  | 0.045 (<10^-18^) |  | 0.117 (<10^-4^) |  | 0.149 (<10^-6^) |
| ∆*CpG*_O/E_ | 0.036 (<10^-11^) |  | 0.002 (0.648) |  | 0.082 (<10^-2^) |  | 0.070 (0.014) |

^a^ “*d*”, intergenic distance; “*#CTCF*”, number of overlapping CTCF-binding sites; “*∆CpG*_O/E_”, difference in upstream DNA methylation.

^b^ *P* values show the probabilities of the observations under the hypothesis of no correlation.

^c^ Spearman’s partial correlation coefficient ρ_p_ is computed by controlling for the other two genomic properties listed in ^a^

**Table S3.** Rank correlations (ρ) and partial rank correlations (ρ_p_) of the examined genomic properties with the divergence time of the adjacent paralogous genes. *#CTCF* was redefined by joint ChIP-seq peaks.

| Genomic properties ^a^ | *d*_S_ | | |  | *T*_phy_ | | |
| --- | --- | --- | --- | --- | --- | --- | --- |
|  | ρ (*P*-value) ^b^ |  | ρ_p_ (*P*-value) ^b,c^ |  | ρ (*P*-value) ^b^ |  | ρ_p_ (*P*-value) ^b,c^ |
| **All adjacent paralogs** | | | | | | | |
| *d* | 0.139 (<10^-5^) |  | -0.086 (<10^-2^) |  | 0.141 (<10^-6^) |  | -0.132 (<10^-5^) |
| *#CTCF* | 0.293 (<10^-22^) |  | 0.272 (<10^-20^) |  | 0.308 (<10^-28^) |  | 0.302 (<10^-28^) |
| ∆*CpG*_O/E_ | 0.097 (<10^-2^) |  | 0.088 (<10^-2^) |  | 0.084 (<10^-2^) |  | 0.069 (0.015) |
| **Adjacent paralogs associated with GO terms in which  high *#CTCF/d* genes were specifically enriched** | | | | | | | |
| *d* | 0.086 (0.152) |  | -0.238 (<10^-4^) |  | 0.108 (0.072) |  | -0.228 (<10^-4^) |
| *#CTCF* | 0.336 (<10^-7^) |  | 0.387 (<10^-11^) |  | 0.349 (<10^-8^) |  | 0.388 (<10^-11^) |
| ∆*CpG*_O/E_ | 0.122 (0.043) |  | 0.068 (0.586) |  | 0.132 (0.027) |  | 0.076 (0.203) |

^a^ “*d*”, intergenic distance; “*#CTCF*”, number of joint CTCF-binding sites; “*∆CpG*_O/E_”, difference in upstream DNA methylation.

^b^ *P* values show the probabilities of the observations under the hypothesis of no correlation.

^c^ Spearman’s partial correlation coefficient ρ_p_ is computed by controlling for the other two genomic properties listed in ^a^

**Table S4.** Assignment of *T*_phy_

| *T*_phy_ | Most recent  common ancestor | Number of adjacent paralogs pairs |
| --- | --- | --- |
| 1 | *Homo sapiens* | 44 |
| 2 | Homininae | 23 |
| 3 | Hominidae | 10 |
| 4 | Hominoidea | 23 |
| 5 | Catarrhini | 69 |
| 6 | Simiiformes | 59 |
| 7 | Haplorrhini | 12 |
| 8 | Primates | 16 |
| 9 | Euarchontoglires | 19 |
| 10 | Eutheria | 257 |
| 11 | Theria | 93 |
| 12 | Mammalia | 103 |
| 13 | Amniota | 112 |
| 14 | Tetrapoda | 55 |
| 15 | Sarcopterygii | 26 |
| 16 | Euteleostomi | 153 |
| 17 | Vertebrata | 59 |
| 18 | Chordata | 47 |
| 19 | Bilateria | 71 |
| 20 | Opisthokonta | 5 |

**Table S5.** The list of non-cancerous cells from which CTCF-binding sites of the human genome identified by ChIP-seq

|  | Cells | Descriptions |
| --- | --- | --- |
| **Fibroblasts** | | |
|  | AG09309 | Adult toe fibroblast |
|  | AG09319 | Gum tissue fibroblast |
|  | AG10803 | Abdominal skin fibroblast |
|  | AoAF | Aortic adventitial fibroblast |
|  | BJ | Foreskin fibroblast |
|  | HMF | Human mammary fibroblast |
|  | HPAF | Human pulmonary artery fibroblast |
|  | HPF | Human pulmonary fibroblast |
| **Endothelium** | | |
|  | HBMEC | Human brain microvascular endothelium |
| **Epithelium** | | |
|  | HEEpiC | Human esophageal epithelium |
|  | HRE | Human renal epithelium |
|  | SAEC | Small airway epithelium |
| **Lymphoblastoid** | | |
|  | GM06990 | Lymphoblastoid cells |
